# Remote population-based intervention for disruptive behavior at age four: study protocol for a randomized trial of Internet-assisted parent training (Strongest Families Finland-Canada)

**DOI:** 10.1186/1471-2458-13-985

**Published:** 2013-10-21

**Authors:** Patrick J McGrath, Andre Sourander, Patricia Lingley-Pottie, Terja Ristkari, Charles Cunningham, Jukka Huttunen, Katharine Filbert, Minna Aromaa, Penny Corkum, Susanna Hinkka-Yli-Salomäki, Malin Kinnunen, Katja Lampi, Anne Penttinen, Atte Sinokki, Anita Unruh, Jenni Vuorio, Carolyn Watters

**Affiliations:** 1 Centre for Research in Family Health, IWK Health Centre, 5850/5980 University Avenue, P.O. Box 9700, Halifax, Nova Scotia B3K 6R8, Canada; 2 Department of Psychology and Neuroscience, Life Sciences Centre, Dalhousie University, P.O. Box 15000, Halifax, Nova Scotia B3H 4R2, Canada; 3 Department of Child Psychiatry, Clinical Sciences, Medical Faculty, Turku University and Turku University Hospital, 20520, Turku, Finland; 4 Center for Child and Adolescent Mental Health, North Norway (RBUP), University of Tromsø, 9037, Breivika, Norway; 5 Department of Psychiatry, Dalhousie University, 5909 Veterans' Memorial Lane, 8th Floor, Abbie J. Lane Memorial Building, QEII Health Sciences Centre, Halifax, Nova Scotia B3H 2E2, Canada; 6 Strongest Families Institute, 7105 Chebucto Road, Suite 355, Halifax, Nova Scotia B3L 4W8, Canada; 7 Department of Psychiatry and Behavioural Neurosciences, Faculty of Health Science, Michael G. DeGroote School of Medicine, McMaster University, Hamilton, Ontario L8S 4L8, Canada; 8 Outpatient Clinic for Children and Adolescents, Itäinen Pitkäkatu 30, 20700, Turku, Finland; 9 Department of Public Health, University on Turku, FI-20014, Turun Yliopisto, Finland; 10 Dalhousie University, Faculty of Health Professions, Burbidge Building, 5968 College Street, P.O. Box 15000, Halifax, Nova Scotia B3H 4R2, Canada; 11 Dalhousie University, Faculty of Computer Science, 6050 University Avenue, P.O. Box 15000, Halifax, Nova Scotia B3H 4R2, Canada

**Keywords:** Disruptive behaviour disorders, Children and youth, Distance treatment, Health service access, Population-based study, Web-based parent training

## Abstract

**Background:**

Oppositional Defiant Disorder (ODD) is characterized by angry and noncompliant behaviour. It is the most common disruptive behaviour disorder (DBD), with prevalence estimates of 6-9% for preschoolers and is closely linked to several long-term difficulties, including disorders of conduct, mood, anxiety, impulse-control, and substance abuse. ODD in children is related to parental depression, family dysfunction, and impairments in parental work performance. Children displaying early DBDs exhibit more symptoms of greater severity, more frequent offences, and commit more serious crimes later in life. The goal of the Strongest Families™ Finland Canada (SFFC) Smart Website intervention research program is to develop and evaluate an affordable, accessible, effective secondary prevention parent training program for disruptive behaviour in preschoolers to prevent the negative sequelae of ODD. Strongest Families is an 11-session program with two booster sessions that focuses on teaching skills to: strengthen parent–child relationships; reinforce positive behaviour; reduce conflict; manage daily transitions; plan for potentially problematic situations; promote emotional regulation and pro-social behaviour and decrease antisocial behaviour.

**Methods/design:**

This protocol paper describes an ongoing population-based randomized controlled trial (RCT) of high-risk 4 year-olds attending well-child clinics in Turku, Finland and environs to examine the effectiveness of the Strongest Families Smart Website intervention compared to an Education Control condition. Randomization consists of a 1:1 ratio for intervention versus the education group, stratified by the child’s sex. The participants randomized to the intervention group receive access to the Strongest Families Smart Website and weekly telephone coaching sessions. The participants randomized to the Education Control condition receive access to a static website with parenting tips. Children are followed using parental and daycare teacher measures at 6 and 12 months after randomization.

**Discussion:**

The Strongest Families Smart Website intervention is hypothesized to improve parenting skills, reduce child disruptive behaviour, reduce parental distress and improve family functioning. These results will likely inform subsequent investigations, public policy, and early treatment of childhood disruptive behaviour problems.

**Trial registration:**

ClinicalTrials.gov # NCT01750996

## Background

### Overview of childhood disruptive behaviour and parent training

Oppositional Defiant Disorder (ODD) [1] is the most common disruptive behaviour disorder (DBD) [2], with prevalence estimates of 6-9% for preschool children (with a higher percentage displaying symptoms that do not meet diagnostic criteria) [2,3]. ODD is characterized by angry/irritable mood, argumentative/defiant behaviour, and vindictiveness. Population-based birth cohort studies have shown that childhood psychiatric problems are developmental precursors for a wide range of negative outcomes indicating risk of marginalization including peer rejection, school failure, psychopathology, substance abuse and criminality [4], and the prognoses are often poor [5-14]. Approximately half of those children whom have been identified as aggressive with externalizing behaviour at preschool age eventually develop persistent problems [15,16] and there seems to be a developmental trajectory of early onset ODD that leads to Conduct Disorder (CD) in a proportion of these children [7]. In children, ODD has also been linked to parental depression [17,18], family dysfunction [18], and impairments in parental work performance [19].

Disruptive behaviour disorders such as ODD are among the most costly of early childhood psychiatric disorders [7]. In children aged 3–8 years, DBDs cause substantial annual costs [20]. Despite an established knowledge base, few strategies have been developed to prevent this trajectory at an early stage and at the population level.

Many parents react to defiant, oppositional behaviour with an increase in controlling strategies and a decrease in positive responses [17]. Forty years ago, Patterson and Reid described the mechanisms via which this coercive pattern contributes to an escalation in disruptive behaviour and more serious antisocial behaviour [21]. This pattern has been confirmed in longitudinal studies [17,18], and leads to diminishing emotional regulation and poor peer relationships [22-24]. Elgar and colleagues [24] examined 4,184 parents and 6,048 ten to fifteen year-old children and youth enrolled in the 1998 and 2000 cycles of the Canadian National Longitudinal Survey of Children and Youth (NLSCY), and found that parenting style predicted both internalizing and externalizing behaviour in children and youth. Although genetics can play a role in disruptive behaviour [25,26], an important implication of the research points to the possibility that early parenting interventions may have epigenetic effects [27,28] that alter the genetic contribution to child outcomes.

Parent training has been shown to be the most effective approach to the prevention and treatment of disruptive behaviour [29-32] and it represents one of the most well-validated therapeutic techniques [33]. The effectiveness of parent training has been established in small groups [34,35], large groups [36,37], and home-based coach-supported distance formats [38].

Parent training is a heterogeneous mode of treatment [39,40], differing in theoretical orientation, amount of intervention, qualifications of the training administrator, mode of delivery, therapeutic components provided, and targeted recipients (e.g., parent only or with the child receiving therapy). In parent training interventions, parents typically learn to identify, define and observe problem behaviours in new ways, as well as learn strategies to prevent and respond to oppositional behaviour [33]. A recent meta-analytic review of parenting programs found that the following program components were consistently related to larger effects: (1) increasing emotional communication skills and positive parent–child interactions, (2) teaching parents to utilize time-out and about the importance of consistent parenting, and (3) requiring practice of new skills between parents and children during parent training sessions [41].

Despite its promise, traditional parent training programs have significant limitations. In Finnish [42], Canadian [43], and American utilization studies [44], most (i.e., about 80%) children with externalizing problems do not receive timely treatment. Moreover, logistical barriers such as child care, transportation time, work schedules, stigma, or discomfort with services delivered in groups prevent many parents from enrolling in or completing parent training programs [39,45-50]. Lastly, low income, limited education, maternal stress, and parental depression [51], can interfere with program completion, limiting the effectiveness of currently available models for families at greatest risk.

The Finnish universal health care system, with its high participation rate in child check-ups and recent emphasis on psychosocial well-being of families (including parenting), represents an exceptional opportunity to study the outcome of parenting skills programs targeted to families with children who present with high levels of oppositional behaviour problems.

### The Strongest Families Finland Canada (SFFC) Smart Website Intervention Program

Based on the reviewed research, we concluded that an ideal intervention for disruptive behaviour problems should, (1) target behaviour problems emerging in the preschool years, (2) be effective, (3) be affordable enough to be implemented widely, (4) appeal to parents who do not use or have ready access to available, traditional programs, (5) be flexible enough to customize the intervention to meet the child’s and family’s needs, and (6) facilitate an integrated system that includes early identification at the population level and management of difficulties, with follow-up after intervention to maintain the effect.

### Study goals

The goal of the research program is to translate and replicate the Strongest Families™ telephone-based program in a Finnish population trial using a Smart Website delivery system. A population-based randomized controlled trial (RCT) is currently being conducted with 4 year-old children displaying disruptive behaviour. Specific objectives of the SFFC Smart Website intervention research program are to:

(1) develop a collaborative network that builds partnerships among investigators that will inform the content of our existing program by harnessing the advances of technology to customize care using videos, audio-clips and written exercises that demonstrate skill implementation;

(2) develop methods for early identification of challenging behaviour in primary health care in Finland;

(3) evaluate the prevalence of early signs of disruptive behaviour in Finnish 4 year-old children;

(4) develop the Strongest Families Smart Website intervention based on the Strongest Families telephone-based program [38] for the prevention and treatment of disruptive behaviour in 4 year-old children;

(5) evaluate the effectiveness of the Strongest Families Smart Website intervention for the early identification and treatment of disruptive behaviour in 4 year-old children, compared to an Education Control condition; and

(6) examine the possible moderating influence of program utilization using tracking reports of website activities (e.g., time on task, number of screens viewed, sessions completed).

### Study hypotheses

#### Primary hypothesis

The primary hypothesis of this study is that the Strongest Families Smart Website intervention will reduce child disruptive behaviour symptoms and impairment scores on the *The Child Behavior Checklist-Parent Report Form (CBCL) 1½–5 years* of the Achenbach System of Empirically Based Assessment (ASEBA) [52] after treatment and at one year follow-up, compared to the Education Control condition.

#### Secondary hypothesis

To supplement this primary hypothesis, it is also hypothesized that after treatment, and at one year follow-up, those participants randomized to the Strongest Families Smart Website intervention will exhibit improved scores on parenting style (*The Parenting Scale [PS]*) [53,54], parental distress (*Depression Anxiety and Stress Scale Short Form [DASS-21]*) [55], and teacher evaluation of school-related child behaviour scores *(The Child Behavior Checklist-Teacher Report Form [TRF]* of the Achenbach System of Empirically Based Assessment [ASEBA]) [52], compared to the participants randomized to the Education Control condition.

## Methods/design

### Design of the study

The study design is a two parallel group RCT stratified by sex, with 1:1 individual allocation comparing the Education Control condition and the Strongest Families Smart Website intervention (Intervention). Best practice guidelines for conducing RCTs will be followed in accordance with the CONsolidated standards of Reporting Trials statement (CONSORT), European International Conference on Harmonisation (ICH) Good Clinical Practice Guidelines, and Tri-Council Policy Statement: Ethical Conduct for Research Involving Humans (trial registry: ClinicalTrials.gov # NCT01750996).

#### Ethics

Our study protocol was approved by our Research Ethics Boards (i.e., Intermunicipal Hospital District of Southwest Finland Ethics Committee, IWK Health Centre Research Ethics). All data is collected with voluntary consent. The voluntary consent forms were formulated according to guidelines set by the Ethics Committee and they were approved in the process of the Ethics approval.

#### Screening and inclusion criteria

Inclusion criteria for the RCT are as follows: (1) the child meets the screening criteria (i.e., the age of 4 years); (2) native language of Finnish or Swedish for at least one of the parents; (3) residence in any of the participating municipalities; (4) in the screening phase, the child has had behavioural challenges for the last six months (score of 5 points or more on the Conduct subscale of the Strengths and Difficulties Questionnaire [SDQ] [56] and with some perceived problems by the parent in the impact section); (5) the parent has access to a telephone, computer, and an internet connection in their home (a computer with an internet connection is provided to families if needed); (6) and the ability of one of the parents to speak and read Finnish.

Participant recruitment began on October 1^st^, 2011 in Turku, Raisio, Kaarina and Naantali cities located in Southwestern Finland (total population of 254,974 at the end of 2012). On October 1^st^, 2012, seven smaller municipalities were enrolled in the study (total population of 76,915 at the end of 2012) [57].

The participants are selected from the Finnish National Population Register [58]. These participants are mailed a study information package approximately one month in advance of the four-year clinic visit. This package contains a brochure reminding the participants of the upcoming extended child health check-up, information about the study required by the Ethics Regulations, and a health questionnaire containing a Finnish translation of the SDQ [56]. The participants are asked to complete the health questionnaire and are encouraged to bring the completed questionnaire to the clinic. If the questionnaire is absent, the health nurses ask the participants to complete the questionnaire during the appointment. In the event that a participant is not interested in the study, the nurse notes “declined to participate”. All of the families of 4 year-old children attending the check-up receive a small token of appreciation from the study team (i.e., a children’s book). After the appointment, the health nurses mail the completed questionnaires to the study site, data is entered into a study database and the SDQ’s are scored.

Those participants with a child meeting the screening inclusion criteria are enrolled in the next phase of the study. The study staff complete a recruitment telephone call introducing the study in more detail and screening for eligibility. During this telephone call, if the participant expresses their preliminary interest in participation, they are registered in the Smart Website electronic platform called IRIS (Intelligent Research and Intervention Software). If the participant declines to participate or is ineligible for the study, a record of this is entered into the system. If recruitment is successful, the data submitted by study staff generates an automated email trigger from IRIS to the participant containing instructions to set up a password for IRIS. On first login, the participating parents are presented with the online consent form. A separate consent telephone call with the parents is scheduled a few days after the recruitment telephone call. During the consent telephone call, the staff confirms the parents’ online consent, and, if consent is provided, the online baseline measures in IRIS are released to be completed by the participating parent. Once the online baseline questionnaires are completed, IRIS sends a task prompt to the study staff to complete the randomization process.

#### Exclusion criteria

Exclusion criteria for the child are as follows: is not speaking in full sentences; is deaf or blind; has received or is receiving behavioural treatment (i.e., parent training); or has a diagnosis of Autism or a Pervasive Developmental Disorder (PDD), Down’s syndrome, Fetal Alcohol Syndrome, mental retardation, genetic diagnosis that will lead to mental retardation, or a major mental health disorder (e.g., depression, psychosis). Exclusionary criteria for parents include: current involvement with child protection services (i.e., removal of child custody, investigation of child abuse or neglect); child not living at home; a major mental health disorder, physical or other severe illness; and long-term hospital visits or care that would interfere with study participation. The exclusion criteria for the child and family are based on parent report during the recruitment call.

#### Sample size

As per Cohen’s [59] suggestion that standardized effect sizes of 0.20, 0.50 and 0.80 be considered as small (detectable), medium and large, respectively, we expect to observe medium to small incremental effects in the order of 0.30 to 0.35 standard units between the intervention and control groups, accounting for multiple comparisons using hierarchical linear modelling (HLM) analysis. This would require 250 participants per group for the trial, allowing for 30% attrition over time. It is expected that adding data from one year follow-up will increase the power to detect differences. An alpha of 0.05 was chosen on an *a priori* basis for the statistical analyses in order to decrease the probability of type II errors.

#### Randomization

The two randomization sequences (i.e., sex stratification) are generated with a 1:1 ratio (i.e., intervention versus Education Control condition) using a computerized random permuted block sequence generator (Random Allocation Software [60]) with concealed block sizes to ensure study staff blinding. A sequential, double envelope system is utilized to conceal individual placement. The sequential envelopes are labeled and colour-coded according to sex. The delegated study staff complete randomization by selecting the next sequential envelope per applicable stratification, affix the sticker that reveals the condition placement on the participant’s source document and enter the group placement information into IRIS. The next study steps released by IRIS are dependent on the group placement entered; IRIS unlocks the appropriate user interface (i.e., Strongest Families Smart Website intervention with weekly telephone coaching-Intervention condition; Static website with parenting tips-Education Control condition). After randomization, the participants receive an email informing them of the randomization results and a link to the relevant website. In addition, the participants are not restricted from seeking other assistance for problems that they encounter.

### Interventions

#### Risk management

This is a minimal risk trial. Staff are trained in risk management reporting protocols to identify and report any suspicion of abuse and neglect according to the local legal requirements.

#### Website security

The internet traffic between the participant and the website is protected by a HyperText Transfer Protocol Secure (HTTPS) communications protocol. The website is hosted on a secure server maintained by the University of Turku IT Services in Finland.

#### Education control condition

The participants randomized to the Education Control condition receive access to a static website with parenting tips as well as a single 45-minute telephone call with a coach reviewing the parenting tips. The Education Control was chosen as the Control condition as opposed to a waitlist control or standard care for the following reasons: (1) a waitlist control condition does not allow for a long-term follow-up, (2) for the majority of families in the Finnish population, standard care constitutes no care at all, and (3) the Education control was deemed as an ethical alternative for parents with a challenging child.

#### Intervention condition

The participants randomized to the Intervention condition receive a Smart Internet version of the Strongest Families telephone-based program [38,61,62], an 11-session, evidence-based parenting program, that focuses on skills for strengthening parent–child relationships; reinforcing positive behaviour; reducing conflict; managing daily transitions; planning for potentially problematic situations; and promoting pro-social behaviour. The Strongest Families telephone-based program was initially designed to target 3–12 year-old children. For the current study, the program content was modified into a web-based format and in accordance with the developmental level of 4 year-old children. Minor culturally appropriate changes were made throughout the program to accommodate for issues such as differences in the schooling and daycare systems between Finland and Canada.

The participating parents are asked to complete the 11 Strongest Families sessions online. The participants are encouraged to complete one session per week and at times most suitable to their daily life using a computer with an internet connection. Only one parent is the active participant during the program; however, the participant is encouraged to share the program content with the other partner when possible. The child does not take part in the telephone calls or use the website. The participant works through the online material for each session including exercises, instructional videos and skill demonstration audio and video clips. During the sessions, the participants are required to complete specific knowledge-based and experience-related questions. The participants are encouraged to complete each session in approximately one week after which the session concludes with a weekly telephone call with a trained coach (with health care professional certification) who provides support, responds to participants’ questions and reviews successful implementation of the program skills. If session skill adoption is considered adequate based on the coach’s professional experience, the next session is introduced at the end of the telephone call. Otherwise, the participant is encouraged to spend additional time with session content to acquire a necessary skill level.

#### Description of strongest families components

The Strongest Families Smart Website intervention has four components. First, the parenting skills curriculum is based on our Strongest Families telephone-based program, an approach derived from programs developed by members of this team [36,37]. There are also two booster sessions to encourage maintenance of skills and outcome gain (i.e., 2–3 months and 4–5 months after intervention). Second, it is a personalized website that tracks and uses activities and interactions to modify the intervention. Each participant’s interactions with the website are personalized by using such factors as the child’s name, child’s problems and strengths, and preferred activities. Each weekly session’s examples and homework tryout pages are populated with the personalized content [63]. The participants are reminded of upcoming appointments or prompted if they had visited the website infrequently. Third, during the initial 11-week phase and booster sessions, the participants are assisted and monitored on their progress by a coach. The coach contacts the participant each week by telephone (45-minute call), reviews their progress, introduces new skills, facilitates the solution of problems, and provides support and encouragement. Prior to each session, the coach reviews the participant’s use of the website and any automatic messages sent to the participants.

#### Treatment fidelity

In order to ensure treatment fidelity, a number of steps are followed, including the recording of telephone calls, strict adherence to standard study protocol procedures, cultural adaptation of the program material to Finnish, and training with coaches. Finnish coaches are health care professionals or semi-professionals with a background in children’s services. They are trained by an experienced Strongest Families clinician to ensure proper application of the program in Finland. Specifically, in addition to ongoing coaches’ meetings to discuss cases conducted initially every two weeks and then once a month with the Canadian team, three training and question/answer sessions for the Finnish group are conducted to ensure correct administration of the Strongest Families Smart Website intervention: (1) one week of intensive program content review, (2) one week of revised curriculum and research protocol training and regulation review per Good Clinical Practice according to the ICH guidelines, and (3) a final process review after the RCT had begun. The Finland study team has weekly study meetings, and coach supervision meetings are held weekly both individually as well as with all of the coaches to review and discuss the cases. All of the telephone calls are recorded and a portion are audited by the Finland coach supervisor and scored for competency evaluation. If scores do not reach competency, additional training is completed with the coach using additional telephone call evaluations.

#### Trial status

**Figure 1:**
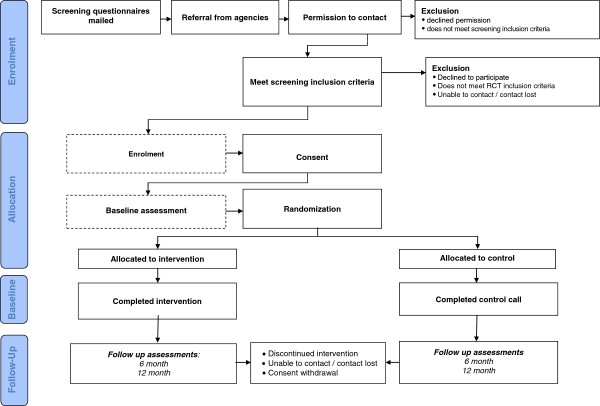
**SFFC Smart Website Intervention Consort Flow Diagram.** (Flow Diagram of the progress through the phases of the SFFC RCT [i.e., enrollment, intervention allocation, baseline, and follow-up]).

Recruitment for the RCT is currently underway, with 337 participants on August 27^th^, 2013. The Strongest Families Smart Website-Finland was launched in January, 2012. We aim to recruit a sample of 500 families and to complete the one year follow-up in 2015 (see Figure 1 for SFFC Smart Website Intervention Consort Flow Diagram).

### Measures

The child and parent applicable measures are completed online using secured access to the Strongest Families Smart Website intervention. The teachers are asked to complete the SDQ and TRF on paper and submit the completed questionnaires to the study team. With the exception of the screening measure, all post-screening measures are administered at baseline, at 6 months and at 12 month follow-up.

#### Screening measure

Screening for the RCT is conducted using the *Strengths and Difficulties Questionnaire* (SDQ) [56]. The SDQ is a brief behavioural screening questionnaire for use with 3–16 year-old children and adolescents. The measure is composed of 25 items that are divided between 5 scales: (1) emotional symptoms, (2) conduct problems, (3) hyperactivity/inattention, (4) peer relationship problems, and (5) prosocial behaviour. Research has found that the SDQ displayed adequate validity in Finnish samples of school-aged children and adolescents, with one study of Finnish school-aged children and adolescents reporting inter-rater agreement for total scores on the parent, teacher, and self-report measures of 0.38-0.44 and internal consistency of 0.71 [64,65]. Another study of Finnish adolescents found correlations between the items and their respective subscales ranging from moderate to high (r = 0.47–0.73), and subscale internal consistency ranging from alpha = 0.53–0.71 [66]. The conduct problem scale of the SDQ is used for screening purposes. Recent research with the parent-rated SDQ in preschool children found that the measure has internal consistency of alpha = 0.58 for the conduct problems scale and acceptable concurrent validity [67].

#### Demographics

Background information on participant native language, marital and socioeconomic status (SES), parental education, and child developmental and medical history is collected.

#### Primary outcome measure

The *Externalizing subscale of the Child Behavior Checklist-Parent Report Form (CBCL) for ages 1½-5* of the Achenbach System of Empirically Based Assessment (ASEBA) [52], the most widely employed, validated and age-normed measure of disruptive behaviour [68-74], is used as the primary outcome measure. The Externalizing subscale of the CBCL consists of 34-items that measure both the angry, irritable, defiant, uncooperative behaviour associated with ODD as well as a broader range of closely related externalizing problems [52]. The CBCL has good test-retest reliability (e.g., 0.81) and criterion validity (e.g., 0.56-0.87) [52].

#### Secondary outcome measures

##### Parenting skills

*The Parenting Scale (PS)*[53,54] is used to measure parenting skills. The PS is a 30-item parent/caregiver report for children ages 1–12 years. It measures parenting and discipline styles, particularly those that are found to be related to the development and/or maintenance of child disruptive behaviour problems. The PS has adequate internal consistency (e.g., 0.84) and convergent validity (e.g., 0.53) [54].

##### Parent conflict

The *Parent Problem Checklist (PPC)* is a 16-item scale measuring parental conflict. Interparental conflict is recognized as a risk factor for many childhood behavioural and emotional problems. This scale has good reliability and validity. High scores on the PPC have been associated with parental reports of increased child problems, general conflict, and lower marital satisfaction [75].

##### Parent’s sense of coherence

The *Sense of Coherence Scale (SOC-13)* is used to assess parents’ sense of coherence (i.e., a global view of the world and individual environment as comprehensible, manageable, and meaningful) [76,77]. The SOC-13 consists of 13 questions with two anchoring phrases. The questions are rated between 1 and 7 on a Likert-type scale, with five items reverse-scored. The sum of all items yields a score from 13 to 91. The reliability and validity of the scale have been established in many studies, and the score of the SOC-13 has been found to correlate positively with various aspects of health and well-being, and negatively with perceived stressors and poor coping skills [76,78]. A systematic review examining 127 studies using SOC-13 found Cronbach alphas ranging from 0.70 to 0.92 and means ranging from 35.39 (SD 0.10) to 77.60 (SD 13.80) points [77].

##### Parental distress

The *Depression Anxiety and Stress Scale Short Form (DASS-21)*[55] is used to measure parental distress. It consists of three subscales (i.e., depression, anxiety and stress) combined into a measure of general distress [79]. The DASS-21 demonstrates strong internal consistency (e.g., 0.93 for the total scale) and adequate construct validity (e.g., 0.69 for the total scale) [80] and is sensitive to the effects of parenting interventions [81-83].

##### Child’s callous and unemotional traits

The *Inventory of Callous-Unemotional Traits (ICU)* is a 24-item questionnaire designed to provide a comprehensive assessment of traits that have been proven to be important for designating a distinct subgroup of antisocial and aggressive youth. The ICU has acceptable internal consistency (e.g., 0.77) and convergent validity (e.g., 0.19-0.44) [84,85].

#### Moderator measure

##### Parent attention-deficit/hyperactivity symptoms

The *Barkley Adult ADHD Rating Scale–IV (BAARS-IV-Quick Screen)*[86] is used to determine whether adult attention-deficit/hyperactivity disorder (ADHD) symptoms moderate the response of parents to the intervention [87]. This 14-item measure takes 3–5 minutes to administer and is linked to DSM-IV diagnostic criteria. It is a reliable and valid measure of current and childhood ADHD symptoms [86].

##### Program utilization measure

Each participant’s time on the website is downloaded using appropriate time-out values and the percentage of primary screens the participants observed. Program satisfaction and therapeutic alliance questions are released at the end of treatment for the intervention group only. Program satisfaction is also measured at 12 months.

##### Quality assurance

All aspects of the trial (e.g., data collection, telephone calls to the participants) are monitored to ensure that data is valid. Staff are centralized in a small call centre and carefully supervised to ensure the system’s integrity.

### Data management and analysis

#### Statistical analyses

Latent growth analysis, mixed effects regression, structured covariance analysis, or hierarchical linear modeling (HLM) will be used. The primary analysis will model the pattern of differences between the groups using growth curve analysis and the statistical software MLwinN [88]. With the primary outcome of CBCL externalizing scores, the analysis will treat repeated measures within individuals as Level 1, and differences in the coefficients between the Strongest Families Smart Website intervention and Education Control conditions as Level 2. In effect, the approach will be to estimate parameters of growth (i.e., the slope of the outcome scores over time) for each individual, and compare these between the Strongest Families Smart Website intervention and Education Control conditions. In addition to comparing trajectories of response among children allocated to the two arms of the trial, net differences in response will be estimated by transposing the intercept to the end of the trial [89]. This will allow a formal test of outcome differences. In addition, aggregate anonymous analysis of the characteristics of eligible children whose families did not consent to participate in the trial will be conducted. This will be important in understanding to whom the results of the trial can be generalized. The next step will be to test the possible moderating influence of program utilization using tracking reports of website activities (e.g., time on task, number of screens viewed, sessions completed).

## Discussion

Disruptive behaviour disorders, such as ODD, are among the most costly of early childhood psychiatric disorders. We expect to have an efficient method to improve access to parenting services that are conveniently delivered to families in the comfort and privacy of their home, removing typical access barriers [39,45-50]. The intervention builds on earlier RCTs, as well as the knowledge of decision makers and parents about what will work in the real world environment. The Strongest Families Smart Website intervention developed through the SFFC program was designed to reduce the prevalence of child disruptive behaviour symptoms through early detection and accessible treatment using this innovative web-based delivery system. Our consumer preference studies [49,50,61,90-92] and our experience with Strongest Families [92] show that personalized coaching is critical to the success of the program.

We are conducting a population-based RCT study of 4 year-olds exhibiting elevated levels of ODD to examine a preventative intervention in the real world rather than in a more controlled situation. The information collected from this study will provide a detailed understanding of how the Strongest Families Smart Website intervention functions, how it can be improved, translated and disseminated. It is anticipated that these results will inform subsequent investigations, public policy, and the treatment of childhood disruptive behaviour problems.

### Strengths and implications

Few interventions to date have employed a population sample and a solid understanding of long-term treatment effects has not been achieved. Importantly, few studies have examined interventions that incorporate the power of interactive web technology to provide a personalized and sustainable intervention for the public health system. This trial will allow for many individuals, including those at greatest risk (e.g., isolated and low income families) who often do not obtain services, to receive evidence-based specialist care. In doing so, this trial will extend the research supporting the effectiveness of parent training for the treatment of ODD by conducting a longitudinal evaluation of an evidence-based, family-oriented web intervention in a Finnish population sample. The incorporation of a Finnish sample allows for a multitude of data to be gathered, as over 99% of the families participate in the health check-ups in maternity and well-baby clinics when invited [93].

## Abbreviations

ADHD: Attention-Deficit/Hyperactivity Disorder; ASEBA: Achenbach System of Empirically Based Assessment; BAARS-IV-Quick Screen: Barkley Adult ADHD Rating Scale–IV; CBCL: Child Behavior Checklist; CD: Conduct Disorder; CONSORT: CONsolidated Standards Of Reporting Trials Statement; DASS-21: Depression Anxiety and Stress Scale Short Form; DBD: Disruptive Behaviour Disorder; HLM: Hierarchical Linear Modelling; HTTPS: HyperText Transfer Protocol Secure; ICH: International Conference on Harmonisation; ICU: Inventory of Callous-Unemotional Traits; IRIS: Intelligent Research and Intervention Software; NLSCY: National Longitudinal Survey of Children and Youth; ODD: Oppositional Defiant Disorder; PDD: Pervasive Developmental Disorder; PPC: Parent Problem Checklist; PS: Parenting Scale; RCT: Randomized Controlled Trial; SDQ: Strengths and Difficulties Questionnaire; SFFC: Strongest Families Finland Canada; SOC-13: Sense of Coherence Scale; TRF: Teacher Report Form.

## Competing interests

PM may benefit indirectly from the study as he holds the trademark and copyright rights outside of Finland for the Strongest Families materials.

## Authors’ contributions

PM and AS led the study, were responsible for securing funding, and made substantive contributions to conceptualization and design of the study, as well as the writing and revision process. PM has given final approval of the manuscript. MA contributed to the planning of the study and manuscript revision. PC and AU assisted in the conceptualization and writing process, and have been involved in the oversight of data collection. PC and AU will also be involved in the writing and publication process. CW assisted in the conceptualization of the study and provided guidance in the use of computers in health care. All of the authors have contributed to the conceptualization, design and coordination of the study and helped to draft the manuscript. All of the authors have read and approved the final manuscript.

## Pre-publication history

The pre-publication history for this paper can be accessed here:

http://www.biomedcentral.com/1471-2458/13/985/prepub
